# The Importance of Synergy between Deep Inspirations and Fluidization in Reversing Airway Closure

**DOI:** 10.1371/journal.pone.0048552

**Published:** 2012-11-08

**Authors:** Graham M. Donovan, James Sneyd, Merryn H. Tawhai

**Affiliations:** 1 Department of Mathematics, University of Auckland, Auckland, New Zealand; 2 Auckland Bioengineering Institute, University of Auckland, Auckland, New Zealand; University of Pittsburgh, United States of America

## Abstract

Deep inspirations (DIs) and airway smooth muscle fluidization are two widely studied phenomena in asthma research, particularly for their ability (or inability) to counteract severe airway constriction. For example, DIs have been shown effectively to reverse airway constriction in normal subjects, but this is impaired in asthmatics. Fluidization is a connected phenomenon, wherein the ability of airway smooth muscle (ASM, which surrounds and constricts the airways) to exert force is decreased by applied strain. A maneuver which sufficiently strains the ASM, then, such as a DI, is thought to reduce the force generating capacity of the muscle via fluidization and hence reverse or prevent airway constriction. Understanding these two phenomena is considered key to understanding the pathophysiology of asthma and airway hyper-responsiveness, and while both have been extensively studied, the mechanism by which DIs fail in asthmatics remains elusive. Here we show for the first time the synergistic interaction between DIs and fluidization which allows the combination to provide near complete reversal of airway closure where neither is effective alone. This relies not just on the traditional model of airway bistability between open and closed states, but also the critical addition of previously-unknown oscillatory and chaotic dynamics. It also allows us to explore the types of subtle change which can cause this interaction to fail, and thus could provide the missing link to explain DI failure in asthmatics.

## Introduction

Deep inspirations (DIs) and airway smooth muscle fluidization are two phenomena which have the potential to counteract severe airway constriction. This is of particular interest as this mediating effect is often found in normal subjects, but impaired in asthmatics (i.e. [1], [2]). Thus understanding their impairment may be central to understanding the pathophysiology of asthma. We consider the interactions between DIs and fluidization, and show that synergies between the two can be critical for effective reversal of severe airway constriction. As such, failure of this interaction may explain the impairment of DI effectiveness in asthmatics. Moreover, our method of analysis lends itself naturally to exploring other modes of failure, such as a reduction in parenchymal tethering effectiveness.

The effect of DIs has been the subject of extensive research, with many studies drawing a distinction between asthmatic and normal subjects on the basis of their response to DI. For example it has been shown that DIs are protective against bronchoconstriction in normals, whereas they fail to limit bronchoconstriction in asthmatics. Normal subjects who are prevented from taking DIs develop a bronchoconstrictive response similar to that seen in asthmatics [3]–[5] and DIs fail to limit bronchoconstriction in asthmatics, where they are effective in normals [6], [7]. A distinction is drawn between a bronchoprotective effect, wherein DIs are taken prior to constriction (and limit subsequent constriction), and bronchodilation, where DIs are taken during constriction (and dilate constricted airways). In addition to the failure of bronchoprotection, asthmatics also display limited bronchodilation due to DI as compared to normals [1], [2].

Fluidization is the response of biological tissues in response to oscillatory or transient strain, typically characterized by a reduction in stiffness, exerted force, and an increase in hysteresivity (i.e. [8], [9]), and as such has been suggested as a potentially powerful mechanism for countering bronchoconstriction. In this work we will consider fluidization of ASM in response to the transient strain of a deep inspiration, which renders the muscle less able to generate constricting force. As such, fluidization in response to a DI is one potential route to bronchodilation, and interactions between the two are a potentially crucial area for understanding the effectiveness (or failure) of the combination in counteracting airway constriction.

We explore the interactions between DIs and fluidization by considering a minimal new mathematical model of a single airway, based on a combination of canonical models in the field. The constituent parts are the Lambert model [10] for the passive stiffness of the airway wall itself; the Lai-Fook model [11], describing the so-called *tethering* forces external to the airway wall from the lung parenchyma; the Laplace law, describing changes in the constricting pressure as the airway narrows; and a ring of activated airway smooth muscle surrounding and constricting the airway. These models are well-established and many studies exist combining some elements, for example Macklem's combination of the Laplace law and linear parenchyma [12], Affonce & Lutchen's study combining airway wall mechanics with linear parenchyma [13], a series of papers considering the combination of the Lambert model and airflow [14]–[16] and the mechanics study of Moreno *et al.* [17]. Similar ideas have also been incorporated into experimental control [18], [19]. Bistability also appears in the terminal airway model of Anafi and Wilson [20], and in the extension to an airway tree by Venegas *et al.* [21], though it is important to note that it is driven by flow, which does not appear in this model. (See ‘Discussion’ for more on this point.)

Such models are often combined and in the process solved iteratively (i.e. [21], [22]), for example first calculating airway radius as a function of transmural pressure using the Lambert model, followed by the calculation of transmural pressure as a function of radius due to the combination of the Laplace law and Lai-Fook parenchymal tethering model. Instead of this two-step approach, we opt to describe the combination of the models as an iterated map, removing the intermediate step of calculating the transmural pressure and instead taking an initial airway radius as input and providing a new, updated radius as output. Thus the airway map, formulated fully in ‘Methods’, may be understood in the following basic form
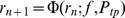
(1)where 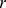 is the airway radius, and 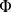 maps one value of r to another; the subscripts denote the iteration of the map. The map depends parametrically on the airway smooth muscle force 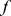 and tethering coefficient (or transpulmonary pressure) 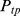; the former describes the force attempting to constrict the airway, and the latter is the coefficient of the restoring, tethering forces attempting to hold it open. (We adopt the term ‘tethering coefficient’ to describe generically the pressure-dependent coefficient of the increase in tethering force due to airway constriction. Please see ‘Methods’ and Eq. 4 for a more detailed discussion of this coefficient.)

One essential idea in the study of airway dynamics is the concept of hysteresis and a bistability between open and closed states, which has been previously explored by a number of authors (i.e. [10], [13], [20], [21]). This is a central idea in this field in that it provides a mechanism which might account for clustered ventilation defects, wherein some portions of a constricted lung are severely constricted, while other regions are normally- or even hyper-ventilated. The airway map exhibits not only the previously studied bistability but also oscillatory dynamics, and in some regimes undergoes a period-doubling cascade leading to chaos [23]. While chaotic behavior has been found in particle mixing in the lung (i.e. [24]) and a coupled tree of airways [21], [25] where the dynamics are driven by flow, we show here for the first time that it also occurs in a minimal model of an individual airway in isolation, without flow. Moreover, these map dynamics are central to the synergistic interactions between DIs and fluidization which make the combination so potent.

## Results

**Figure 1:**
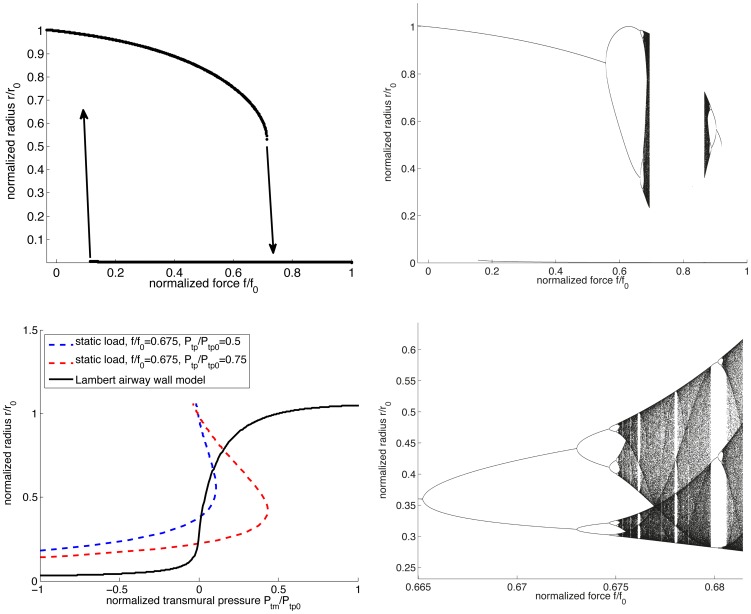
Bistability to chaos. One-dimensional bifurcation diagrams for the airway map with ASM force varied and 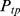 fixed, and static load curves. Top left panel: the traditional view of bistability and hysteresis found when 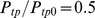. Beginning at zero force, the airway is open with increasing force up to approximately 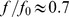 where a sharp transition to closure occurs. Retracing from the closed state by decreasing applied force results in the closed state persisting all the way back to 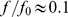. In this range the open and closed states both exist. Top right: period-doubling route to chaos found when 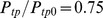. Bottom left: static load curves and Lambert airway wall model. The solid black curve gives the Lambert airway wall mode [10] for an order 5 airway. The dashed lines give the static load determined by Eq. 3 for each of the cases in the upper panels – the blue curve corresponds to the simple bistability of the 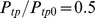 case in the top left, while the red curve represents the case with the oscillations and chaos of the 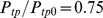 case in the top right (see text). Bottom right: detailed view of top right panel.

With the airway model formulated as an iterated map describing airway radius, we have the tools necessary to study its behavior as important parameters are varied; in this case we are principally interested in the strength of ASM constriction, as controlled by the parameter 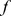, and the strength of parenchymal tethering, as controlled by the parameter 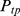 (see ‘Methods’). We begin by holding the tethering coefficient (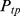) fixed and varying the applied ASM force (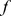). Initially, for 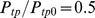, we find the typical well-known bistability and hysteresis loop (Fig. 1, a), allowing for both closed and open states to coexist for some range of 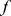 (i.e. [13], here for 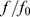 ranging from approximately 0.1 to 0.7). Beginning at zero force, the airway is open, and stays open with force increasing up to approximately 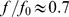 where a sharp transition to closure occurs. Retracing from the closed state by decreasing applied force results in the closed state persisting all the way back to 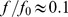. In this range the open and closed states both exist. However, upon increasing the tethering strength to 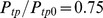, we find not only this bistability, but also an oscillatory regime beginning around 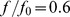 leading into a period-doubling cascade to chaos [23] (Fig. 1, b). A zoom to show detail of this route to chaos is given in panel d. The variations observed in the oscillatory and chaotic regime are also significant, ranging from an entirely open airway to one quite severely constricted.

One way to attempt to address the origin of these behaviors is to look at the balance of the static loads between 1) the airway wall itself and 2) the combination of the ASM force and parenchymal tethering. These static load curves are given in Fig. 1, panel c, for each of the two cases above, specifically at 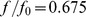 where the behavior is either bistable or chaotic (depending on the choice of 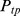). The solid black curve gives the Lambert airway wall model [10], and the dashed curves the ASM/parenchyma static load (blue for the bistable case, red in the chaotic case). In principle, from this analysis, one might expect each situation to have three solutions, one at each point of intersection near zero transmural pressure, and another at the intersection representing closure near zero radius and large negative transmural pressure (not shown). However, this is not the case and demonstrates the need to consider the problem as a map, as otherwise the stability is ignored and important dynamics can be overlooked. In fact in the case of the blue curve there are two stable and one unstable equilibria, as shown in panel a, and for the red curve there are no stable equilibria at all (panels b and d).

**Figure 2:**
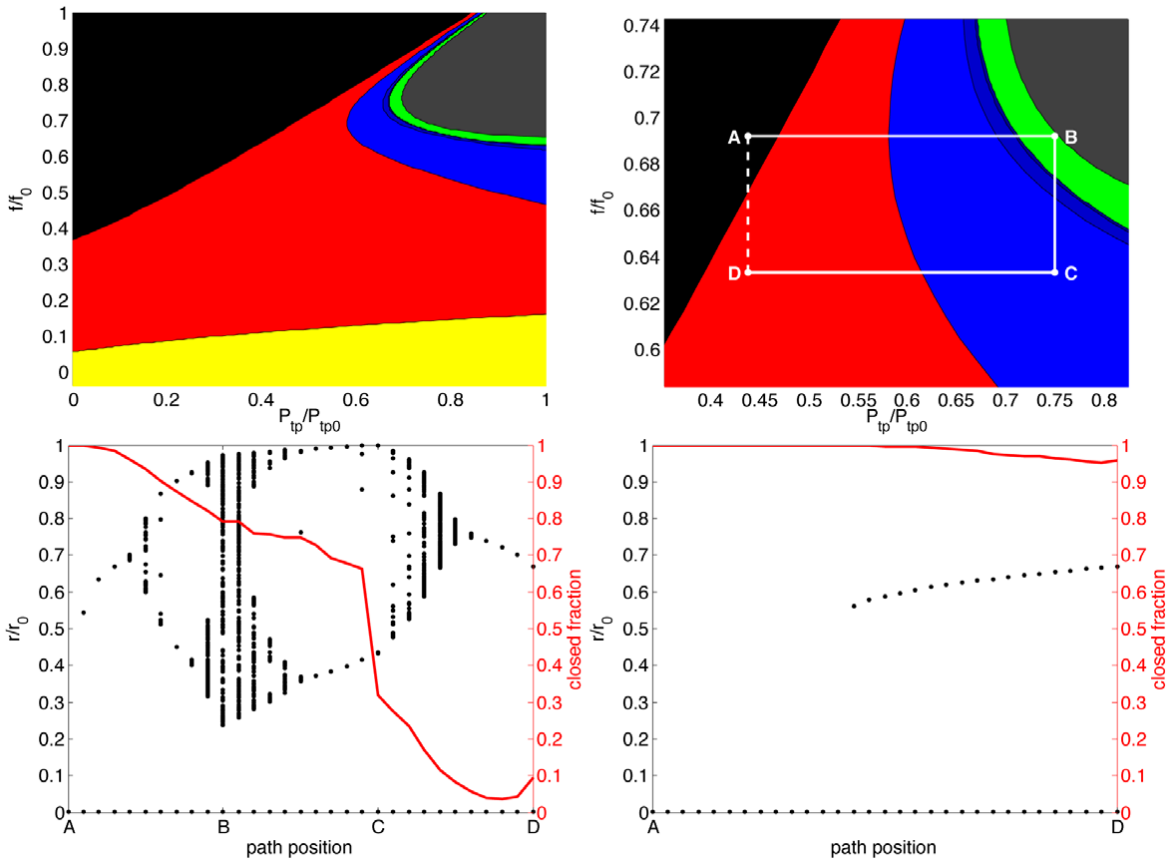
2D bifurcation sets and pseudo-dynamics. Top left: 2D bifurcation set for the airway map, color-coded by behavior. Yellow: one fixed point, airway open; red: bistability; black: one fixed point, airway closed; blue: oscillations, with darker shades for longer period oscillations; green: chaos; grey: three fixed points, all near closure. Top right: detail showing the path of DI pseudo-dynamics. Bottom left: Pseudo-dynamics for full DI path (A-B-C-D). Left axis, black dots: airway radii for 1000 samples, each dot corresponding to an airway at each path position. Right axis, red line: fraction of closed airways in the sample. Throughout the maneuver airways gradually open, with a near-complete opening of closed airways at the conclusion. Bottom right: Pseudo-dynamics for direct force reduction without DI (A–D). Along this path, without the DI, near-total closure persists.

While these examples demonstrate the existence of rich dynamics, the dependence upon tethering coefficient 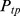 calls for a two-parameter bifurcation study to understand the influence of both the constricting and restoring forces. Varying both 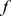 and 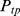 now simultaneously, we can no longer plot the value of each point obtained from the map but instead classify the results into categories as follows: one stable fixed point, airway open; one fixed point, airway closed; bistability; oscillations, period 2; oscillations, period 4; oscillations, period 8; chaos. Each of these cases occur in one or more regions of the 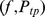-plane. By analysing the map we can find the boundaries between each of these regions, given in Fig. 2 (a). Color coding corresponding to the categories above is given in the figure legend.

We can clearly see that for values of 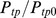 less than approximately 0.58, the traditional view exists: a single, open, fixed point for small values of 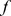, followed by a region of bistability, and finally for sufficiently large 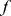 only a single closed state (as in Fig. 1 (a)). Right of this line, however, the new behavior emerges. Initially period-two oscillations emerge, then progressing through a period-doubling cascade to chaos. At the end of chaos, the closed state again prevails; (we have colored this grey, rather than black, to reflect that while empirically only the closed state exists, formally there are three fixed points here, each sufficiently small as to be considered closed states.) While the existence of this route to chaos in this model is new and interesting in its own right, most importantly it explains the synergistic interaction between DIs and fluidization which result in effective bronchodilation.

Consider the path in parameter space marked out by the points A, B, C and D (Fig. 2, (b)), and suppose that we begin with a population of severely constricted airways at A – only the single closed state exists here. This point we think of as analogous to a severely constricted lung. The path of a DI combined with fluidization, then, might be approximated as A-B-C-D, with an increase in 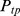 as the DI is drawn (to B), a decrease in ASM force due to fluidization induced by the DI strain (to C), and a decrease again in pressure as the breath is exhaled (to D). As D is in the bistable region, in principle here our population may now be entirely closed, entirely open, or anywhere in between. What is important is the dependence of this closed fraction *on the path taken*. The pseudo-dynamics of a population of airways along this path (see ‘Methods’) are computed and given in Fig. 2 (c), showing the initially closed population beginning to open as the maneuver progresses. Initially a small number of closed airways jump into the open state upon moving into the bistable region, with a gradual increase as the path moves through the period-doubling cascade and into chaos. As the path moves back out of chaos (toward C) and traverses the cascade in reverse, the rate of opening increases dramatically, as can be seen from the red closed fraction line, and continues as the breath is exhaled (to D). At the end of the maneuver, more than 90% of the airways have been reopened. The combination of a DI and fluidization together is thus highly effective at reversing airway closure.

Contrast this with the direct path A–D, with the same reduction in force but without the DI itself; this is equivalent to fluidization alone. The pseudo-dynamics of this path are given in Fig. 2 (d). Here some small fraction of points do move into the open state along the path, but without the progression through the period-doubling cascade and the band of chaos, more than 95% of the airways remain in the closed state. Thus despite beginning and ending at the same points, one can have either a near-complete reopening from closure, or near-complete failure.

Of course, a DI alone without fluidization is equivalent to the path A-B-A. Independent of any opening which occurs along this path, upon return to point A all airways must be in the closed state – this is the only fixed point there, and so the closed fraction must be 1. Thus the airway map demonstrates the much greater power of a DI and fluidization together, as compared to either alone. It is worth noting that the precise location of point B in the chaotic band is not critical; the general requirements for reopening are that the path traverses the oscillatory or chaotic bands, and that the terminal point be in the bistable region. The specific path given here is in this sense generic.

**Figure 3:**
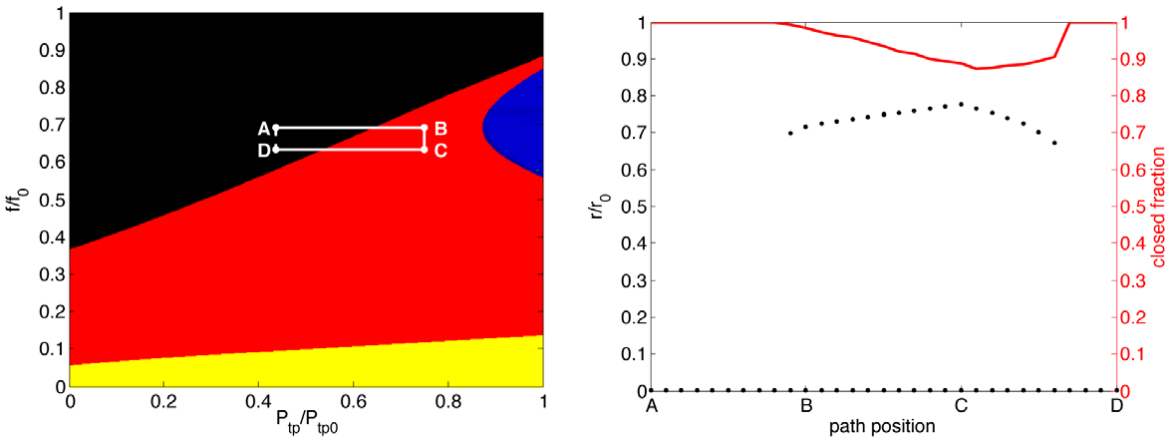
Bifurcation set and DI pseudo-dynamics, with modified parenchymal tethering nonlinearity (see text). By reducing the degree of nonlinear tethering, the DI and ASM fluidization combination fail completely in reversing airway closure. Even transient dilation is limited, with more than 85% of airways remaining in the closed state throughout.

If the effectiveness of a DI is dependent upon synergies with fluidization, then what can this tell us about the possible mechanisms for DI failure in asthmatics? While the map does not in itself suggest a specific distinction between normals and asthmatics, it does raise the prospect that the difference may be quite subtle. Consider, for example, the hypothesis that parenychmal tethering is less effective in asthmatics, relative to normals. One manifestation of this hypothesis could be that the nonlinear coefficient of tethering (see Eq. (4), ‘Methods’) is reduced. As an illustration, we reduce this coefficient from its standard value (1.5, as in [11]) to 0.5 and recompute both the bifurcation sets and DI pseudo-dynamics resulting from this modified model, with the results given in Fig. 3. Now this path begins and ends in the region with only the closed state available; thus the end result of the DI maneuver must be that all airways are closed. It is also instructive to observe that even in the portion of the path which traverses the bistable region, more than 85% of the airways remain closed. Thus the decreased tethering force has eliminated the reversal of airway closure from a DI, and limited even the transient effects. While this arbitrary modification is not directly indicative of the difference between normals and asthmatics, it is an illustration both of the type of subtle phenomena which may account for the difference, and the power of this type of analysis to shed light on the many proposed hypotheses – particularly in light of recent controversy surrounding the role of DIs and fluidization in limiting airway constriction in intact airways; see ‘Discussion’.

## Discussion

Looking at the problem of airway constriction as an iterated map is a new way of analyzing airway behavior. It has yielded critical insight into the relationship between DIs and ASM fluidization, which helps to explain the importance of synergy between the two in reversing airway closure. In addition, it is a powerful new tool for the study of the myriad hypothesized differences between asthmatic and normal subjects. We have illustrated, with a simple example of reduced parenchymal tethering nonlinearity, the possibility of a relatively subtle change significantly altering the DI and fluidization dynamics (such that reversal of several airway constriction no longer results from the DI-fluidization synergy). While it is merely an example, rather than a concrete hypothesis, it does demonstrate the ability of the airway map to quickly and easily test the effect of many such theories.

We have also shown that airway states, including both the previously-known (open, closed, and bistable) and the new states (oscillatory and chaotic), can be found in a model which does not include a force-length relationship, ASM dynamics, or ventilation. The static bistability in a single airway is in keeping with the findings of Affonce and Lutchen [13], who demonstrated the potential of the bistability to contribute to airway hyper-reactivity. The Anafi and Wilson model [20] also exhibits a well-known bistability, which has in common some elements with the model presented here, but also key differences. It remains possible that the Anafi-Wilson terminal airway model would exhibit a similar bistability to that shown here, based on the elastic mechanics alone in the absence of flow; however this has not been shown and remains the subject of speculation. Similarly their terminal airway model was extended by Venegas *et al.* [21] into a symmetrically branching airway tree. This extended model was shown to exhibit chaotic switching driven by parallel flow and the interacting structure of multiple airways. Here we have shown for the first time that such chaotic behavior can occur in a minimal single airway model, even in the absence of ventilation, and moreover that these chaotic and oscillatory dynamics yield a potential explanation of synergy between deep inspirations and fluidization.

The findings here should also be considered in light of recent controversy surrounding the role of DIs and fluidization in bronchodilation, due to discrepancy between tissue strip and excised airway studies [4], [26]–[28]. We have shown here that synergy between fluidization (or, in fact, a reduction in ASM force by any cause) and DI in airway reopening. However, the idea that fluidization may not bear primary responsibility is certainly worth considering. The methods of analysis presented here are useful tools for evaluating other hypothesis as well.

One significant limitation of the airway map model is that no proper attempt is made to account for the dynamics of ASM itself – ASM force is modelled in the simplest possible fashion, as a prescribed, exerted force. As such, ASM fluidization can only be represented by a simple reduction in exerted force, rather than a process occurring over time. This is a significant assumption, as many potentially important phenomena are ascribed to ASM dynamics (i.e. [29]), and we have previously shown the ability of ASM dynamics to modulate transitions between open and closed airway states [22]. While many models of ASM dynamics are available in the literature (i.e. [30]–[33]), modifying the model and analysis presented here to include such effects is as yet an unsolved problem. This remains an important area for future work.

We have assumed throughout that the force-length relationship is constant. Though our formulation easily allows for an approximation to the experimental data (i.e. [34]–[37]), we have not done so at this stage for two reasons. The first is that properly accounting for the force-length relationship requires a full dynamic model, the difficulties with which are discussed in the preceding paragraph. Secondly, under a simple, static force-length relationship (i.e. [22]), the critical behaviour is driven by the force exerted at very short lengths far from the adapted length, which must typically be extrapolated from the experimental data. In the absence of a dynamic model and detailed data at the short end of the force-length curve, the best assumption is a constant relationship. It is interesting to note that bistable behavior does occur in this model in the absence of ASM force-length dependence, much as it has previously been shown that flow and compensatory pathways are not required [13]. Thus there are several mooted mechanisms driving bistability, each of which could plausibly explain observed heterogeneity and patchy ventilation defects when coupled with a suitable organizing principle. However, we have shown more than just a new route to bistability, but also new oscillatory and chaotic dynamics which were previously unknown and lead to a possible explanation of synergy between fluidization and deep inspiration.

## Methods

The 1D continuous map is constructed by combination of the Lambert model [10], which relates airway transmural pressure 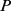 and radius 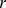 as
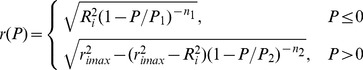
(2)where the parameters 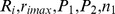 and 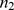 depend upon airway order [22] and are given explicitly at the end of this section. Throughout we have used an order 5 airway; results are similar for other small caliber airways. The transmural pressure 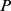 is given by
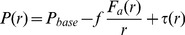
(3)where 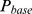 is the base transmural pressure, the second term reflects active muscle force 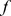 (along with the force-length relationship 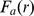) and the Laplace law 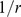 dependence, and the third term is the parenchymal tethering. Following the Lai-Fook model [11] we have
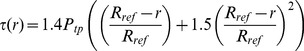
(4)where the reference radius 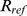 is taken according to Eq. 2 at a transmural pressure of 10 cmH_2_O. The leading coefficient of Eq. 4 bears further discussion. We adopt the notation of Lai-Fook [11], using 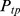 and referring to it as the *tethering coefficient*. In [11] this is the transpulmonary pressure, by way of its connection with the parencyhmal shear modulus 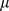, where 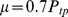. In the formulation of Anafi and Wilson [20] the symbol 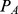 is used instead, in the context of flow-driven behaviour. We adopt the notation of the former as the most natural for a model in the absence of flow.

Thus by substituting Eq. 4 into 3, and then into 2 we obtain the composite function 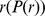 and call this
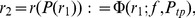
(5)and thus the combined model may be thought of as a 1D iterated map. We include explicitly the parametric dependence on 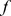 and 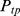 as these are the bifurcation parameters used here. The full explicit form in terms of 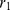 is then given by
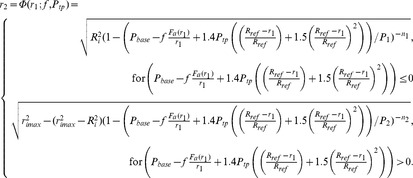
(6)Note that continuity of the piecewise function is ensured by the definition of 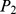 [10], [22]. It is also important to observe that the smooth muscle force 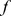 is treated in the simplest possible way, as a prescribed, exerted constant force. In fact, this is an entirely static model and time does not appear at all. While more sophisticated models of ASM are available (i.e. [30]–[32]), including such dynamics significantly complicates the analysis. See ‘Discussion’ for more detail. The baseline transmural pressure 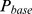 is taken to be 10 cmH_2_O throughout. We assume no force-length relationship, that is 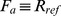. The implications of, and reasons for this assumption are addressed in ‘Discussion’.

The one-dimensional bifurcation diagrams in Fig. 1 are made by brute force iteration of the map, starting from 5 evenly spaced seeds between 0 and 2 mm. If the iterations converge to a fixed point with a tolerance of 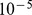 mm, that point is plotted alone; if there is no convergence after 2000 iterations, the last 200 points are all plotted together.

The static load curves of Fig. 1 (c) were created using Eqs. 2, 3 and 4 as follows. The solid curve representing the Lambert airway wall model comes from Eq. 2 alone. The static load curves are obtained by substituting Eq. 4 into 3.

The boundaries of the 2D bifurcation sets in Fig. 2 are computed by the more sophisticated methods used to analyze such maps. For example, boundaries between one fixed point and bistability (yellow-red, and red-black) occur where
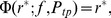
(7)

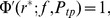
(8)the prime denotes differentiation with respect to 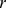 and 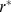 is the fixed point [38]. These, and similar equations for the other boundaries [38], [39] must be solved numerically by a technique known as numerical continuation, which allows solutions to be followed as parameters change [40]. For purposes of classifying fixed points in bifurcation sets (i.e. is the fixed point open or closed?) we use a threshold of 41% of reference radius (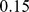 mm for an order 5 airway with 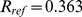 mm).

The pseudo-dynamics in Fig. 2 are computed by sampling 1000 separate and independent airways, each of which begins at point A and make 10 steps along each segment of the path. At each step, each airway is perturbed by an additive Gaussian random variable and then iterated in the map 30 times. This value is then taken to be the new radius for the airway at that step. The additive random variables have zero mean and standard deviation 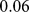 mm, which is 16.6% of 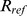 for an order 5 airway.

The parameter values used throughout are 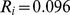 mm, 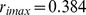 mm, 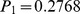 cmH_2_O, 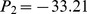 cmH_2_O, 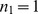, 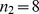, 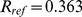 mm, 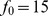 cmH_2_O, and 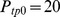 cmH_2_O. These are taken from [22] for an order 5 airway.
